# Emerging Artificial Intelligence (AI) Technologies Used in the Development of Solid Dosage Forms

**DOI:** 10.3390/pharmaceutics14112257

**Published:** 2022-10-22

**Authors:** Junhuang Jiang, Xiangyu Ma, Defang Ouyang, Robert O. Williams

**Affiliations:** 1 Division of Molecular Pharmaceutics and Drug Delivery, College of Pharmacy, The University of Texas at Austin, Austin, TX 78712, USA; 2 Global Investment Research, Goldman Sachs, New York, NY 10282, USA; 3 State Key Laboratory of Quality Research in Chinese Medicine, Institute of Chinese Medical Sciences (ICMS), University of Macau, Macau 999078, China

**Keywords:** solid dosage formulation, artificial intelligence, machine learning, deep learning

## Abstract

Artificial Intelligence (AI)-based formulation development is a promising approach for facilitating the drug product development process. AI is a versatile tool that contains multiple algorithms that can be applied in various circumstances. Solid dosage forms, represented by tablets, capsules, powder, granules, etc., are among the most widely used administration methods. During the product development process, multiple factors including critical material attributes (CMAs) and processing parameters can affect product properties, such as dissolution rates, physical and chemical stabilities, particle size distribution, and the aerosol performance of the dry powder. However, the conventional trial-and-error approach for product development is inefficient, laborious, and time-consuming. AI has been recently recognized as an emerging and cutting-edge tool for pharmaceutical formulation development which has gained much attention. This review provides the following insights: (1) a general introduction of AI in the pharmaceutical sciences and principal guidance from the regulatory agencies, (2) approaches to generating a database for solid dosage formulations, (3) insight on data preparation and processing, (4) a brief introduction to and comparisons of AI algorithms, and (5) information on applications and case studies of AI as applied to solid dosage forms. In addition, the powerful technique known as deep learning-based image analytics will be discussed along with its pharmaceutical applications. By applying emerging AI technology, scientists and researchers can better understand and predict the properties of drug formulations to facilitate more efficient drug product development processes.

## 1. Introduction

Active pharmaceutical ingredients (APIs) are mainly formulated into solid-state forms and subsequently delivered to patients through different routes of administration. Among various drug products on the market, solid dosage forms provide the most popular administration method [1]. Solid dosage forms consist of one or multiple APIs and suitable excipients, including binders, antioxidants, disintegrants, stabilizers, granulating agents, etc. [1]. The development of solid dosage forms is usually complex and requires a deep understanding of information such as physicochemical properties and pharmacokinetic/pharmacodynamic modeling (PK/PD). The development contains several processes, including pre-formulation, drug product development, and manufacturing [2,3]. During the formulation development process, a large number of factors must be considered, including solubility, polymorph, stability, excipient compatibility, analytical method development and validation, dissolution, bioavailability, manufacturing, and scale-up [4]. Low aqueous solubility, one of the most critical challenges during formulation development, can be represented by biopharmaceutical classification system (BCS) classes II and IV [5]. It has been reported that approximately 40% of commercial drug products and 90% of drugs in development are defined as poorly water-soluble [6]. Some other challenges in the formulation development process include low powder flowability [7], a narrow therapeutic window [8], and chemical degradation during the manufacturing process [9]. To address the challenges encountered during formulation development, scientists must perform numerous experiments to attempt to fill the knowledge gap. These experiments are both laborious and time-consuming. Artificial intelligence provides a solution to this problem because it is an efficient, effective approach, which has become more powerful and flexible in recent years [10].

Artificial intelligence (AI) is a process that simulates human intelligence using computers. The concept was first proposed in 1956 during a conference led by Marvin Minsky and John McCarthy [11]. A typical AI workflow consists of four steps: Obtaining and preparing data, AI modeling, simulation, testing, and deployment [12]. Machine learning, a subcategory of AI, is referred to as the process of implementing algorithms and recognizing patterns from the data to facilitate decision-making [13]. Decision-making examples include healthcare operational decisions [14] and decisions for risk forecasts [15,16]. As a subfield of machine learning, deep learning is typically represented by layered-structure algorithms, also known as artificial neural networks (ANN). ANNs, which were inspired by the biological neuron structure in human brains, exhibit more outstanding computational and predictive capability compared to conventional machine learning algorithms [17]. In addition, deep learning has been widely used for multiple applications such as image classification [18], object detection [19], image segmentation [20], natural language processing [21], and medical image analysis [22].

AI-based drug development has been widely applied in the pharmaceutical industry and is considered a potential and powerful strategy compared to the conventional pathway. The AI approach combines multiple disciplines including chemistry, material science, chemical engineering, computer science, computer vision, and machine learning. Pharma 4.0 is a framework for applying novel digital techniques to solve some long-existing obstacles in pharmaceutical manufacturing [23]. In a 2020 publication, Wang et al., illustrated a comprehensive landscape of computational pharmaceutics and “Pharma 4.0” from the perspective of different machine learning models, process simulation, mathematical models, molecular modeling, and physiologically based pharmacokinetic (PBPK) modeling [24]. This publication by Wang et al., also summarized the regulatory requirements, challenges, and future perspectives in pharmaceutical industries [24].

**Figure 1:**
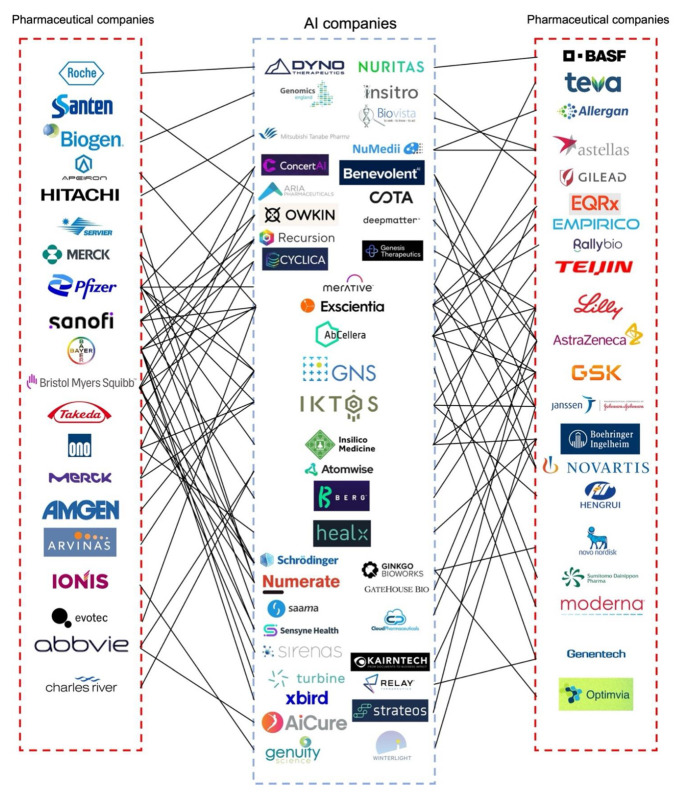
Partnerships between AI and pharmaceutical companies formed for drug product development. This summary gives an overview of the recently reported collaborations between AI and pharmaceutical companies. Most of the partnerships are related to drug discovery and clinical studies. AI and pharmaceutical companies have limited partnerships with regard to formulation development, especially solid dosage forms. Most of the research related to the development of solid dosage forms using AI is conducted in universities. Information in this figure was obtained from the literature [26], company reports, press releases, and the Securities and Exchange Commission (SEC) filing. A full list of key partnerships between AI and pharmaceutical companies and the corresponding references can be found in Supplementary Materials. Figure adapted and updated with permission from reference [26] 2019. Drug Discovery Today. Aria Pharmaceuticals was formally named twoXAR; Merative was formerly named IBM Watson Health.

Due to the rapid development of AI in the pharmaceutical industry, the global AI market for the pharmaceutical industry is expected to reach $1.24 billion in 2022 at a compound annual growth rate of 32.3% [25]. More importantly, pharmaceutical companies have invested in AI companies or formed joint ventures with the goal of developing better drug products and medical devices [26]. Applications of AI have already significantly improved decision-making, research, and clinical trial efficiency to provide benefits for patients, physicians, insurers, and regulators [26]. This trend is continuing with numerous pharmaceutical companies collaborating with AI technology companies and institutions incorporating AI as a tool during product development. For example, Merck & Co and Bayer were both granted the Breakthrough Device Designation from the U.S. Food and Drug Administration (FDA) for artificial intelligence software to be used to support clinical decision-making regarding chronic thromboembolic pulmonary hypertension. Novartis and Pfizer collaborated with the Massachusetts Institute of Technology (MIT) to create the Machine Learning for Pharmaceutical Discovery and Synthesis Consortium to facilitate the design of useful software for the automation of small molecule discovery and synthesis [27]. AstraZeneca entered into a collaboration with Ali Health aiming to expand the drug market in China while using AI to help patients access optimal medicines [28]. The global scale partnerships developed between AI and pharmaceutical companies are summarized in Figure 1. As shown in Figure 1, as of June 2022, a British AI company Exscientia had nine partnerships with pharmaceutical companies, more than all other AI companies, followed by IKTOS, and GNS Healthcare. Additionally, the formation of various consortiums, including the Machine Learning Ledger Orchestration for Drug Discovery (MLLODDY) and the Machine Learning for Pharmaceutical Discovery and Synthesis Consortium (MLDPS), have greatly facilitated the process of drug discovery by creating a more advanced platform without sacrificing the data privacy of the participating companies. Therefore, the increasing formation of partnerships between AI and pharmaceutical companies along with ML-related organizations effectively facilitates the pharmaceutical product development process.

From a regulatory perspective, the U.S. FDA issued the “Artificial Intelligence/Machine Learning (AI/ML)-Based Software as a Medical Device (SaMD) Action Plan” in 2021 and aimed to tailor regulatory oversight and enable the improvement of patients’ lives [29]. In recent years, the FDA has approved several AI-based products such as Idx-DR, OsteoDetect, Guardian Connect System, ContaCT, and FibriCheck. AI-based software is subject to U.S. FDA review based on its risk classification. Class I products, such as the glucose level monitor reader, tend to have the lowest risks. Most AI-based software programs are class II products that typically undergo the 501(k) pathway or De Novo approval. Class III products pose the highest risks and must undergo the whole premarket approval process. In addition, as a regulatory agency, the FDA dictates that the computational methods in pharmaceutics should promote product quality and comply with the quality by design (QbD) strategy [30]. Moreover, quantitative methods and modeling (QMM), which includes physiologically based models, plays an essential role in bioequivalence (BE) assessment and has been increasingly applied by the U.S. FDA [31]. The U.S. FDA and the European Medicine Agency (EMA) show positive inclinations toward model-informed drug discovery and development (MID3) which aim to improve the implementation, standardization, and acceptance rate of the related approaches within drug development and regulatory review [31]. In 2019, the U.S. FDA announced the Knowledge-aided Assessment & Structured Application (KASA) quality assessment system designed to establish algorithms and rules for risk assessment and control and to conduct computer-aided analysis to compare regulatory standards and quality risks across facilities and applications [32].

Solid dosage formulation, one of the most important dosage forms in pharmaceuticals, accounts for over 50% of NMEs (NMEs: New Molecular Entities) continuously, according to the FDA CDER (CDER: Center for Drug Evaluation and Research), due to its many benefits, including shelf stability, patient compliance, ease of transportation, and precise dosage [33]. This review aims to (1) provide formulation scientists with a brief introduction to AI methodology including the different algorithms available, and (2) highlight the emerging AI tools that can be applied in the solid dosage formulation development process.

## 2. Commonly Used Databases

The first step in performing AI-based analysis is to obtain a database. Building a high-quality database is the prerequisite to successfully developing a suitable model for formulation development. There are several conventional pathways for establishing a database for modeling, including using (1) an external database that is available to the public, (2) an internal experimental database that was built prior to the public database, and (3) a database generated by experimental approaches using statistical data collection and analysis methods such as the Design of Experiment (DOE) tool [34]. DOE is a structured method that enables scientists to (1) understand the relationship between multiple factors and responses, (2) determine the interaction between different factors, and (3) optimize the response [35]. The following table is a summary of the open-source databases containing information on solid dosage formulations (Table 1). The table lists the data sources for APIs, excipients, and formulations information separately which is helpful for the build-up of the preliminary dataset. For example, as one of the most popular and widely used chemical information websites, PubChem provides information containing 112 million compounds, 297 million substances, 1.5 million bioassays, 296 million bioactivities, 185 thousand proteins, and 43 million patents from 871 organizations globally. In the U.S. FDA inactive ingredients database, seven fields, including inactive ingredients, routes, dosage forms, CAS numbers, UNII, potency amounts, and potency units, are listed for 9438 inactive ingredients as of August 2022. Drugs@FDA is a database of FDA-approved drugs which contains drug information and biological products that are approved for human use in the U.S. Specifically, the database lists the information on the approved drugs including the active ingredients, strengths, routes, dosage forms, market status, therapeutic equivalence (TE) codes, reference listed drugs (RLD), and reference standards (RS). In addition, for prescription brand-name drugs, the database includes the most recent labeling information approved by the FDA, regulatory information, and FDA staff reviews. By conducting data mining from internal or open-source databases, scientists are able to construct a high-quality dataset for machine learning modeling.

## 3. Data Processing Methods

### 3.1. Tabular Data Processing

After obtaining a raw database from public resources or in-house experimental results, scientists need to process the data before building the models. Some commonly used approaches, including data cleaning, dimension reduction, imbalanced data solutions, and data splitting, are necessary to adjust and then analyze the data. Data cleaning is a processing method used for missing or inaccurate dataset observations. Data cleaning can be implemented by removing the data points or replacing them with mean/median values [48]. However, there are some limitations to removing missing values; for example, the reduction of data size could affect the robustness of the model [49]. Dimensionality reduction is a method to remove less important features in the database which will reduce the model’s complexity and may mitigate overfitting issues. Several dimensionality reduction methods, such as principal component analysis (PCA), low variance filtering, high correlation filtering, and random forest feature selection have been widely used for data processing [50]. Imbalanced data typically refers to the unequal distribution of different classes within the database. For the prediction model using an imbalanced dataset, the prediction metrics results, especially accuracies, are not representative because the model’s overall accuracy tends to be biased towards the majority class regardless of the minority class with small amounts of samples which will lead to poor performance [51]. Moreover, in published literature, researchers tended to bias reporting towards more positive results, and this will lead to an imbalanced database during data mining. To solve this problem, some oversampling and under-sampling strategies, including the Synthetic Minority Oversampling Technique (SMOTE), ADAptive SYNthetic sampling (ADASYN), the Edited Nearest Neighbor (ENN) method, the Condensed Nearest Neighbor method can be implemented to address the imbalance issues [52]. In addition, to evaluate the model performance of imbalanced data, some metrics such as Cohen’s Kappa and Receiving Operating Characteristic (ROC) Area Under the Curve (AUC) are more representative than others [53]. Data splitting is another one of the most critical procedures in data processing. With this procedure, the whole data set will typically be randomized and divided into three subsets: training, validation, and testing. Training datasets are the portion of the data that will be initially fed into the model to teach it how to make a prediction. Validation and test subsets are used for the model’s validation to prevent over-fitting. Conventional ratios for these three subsets are 70%/20%/10% for training, validation, and testing, respectively; however, the ratios also depend on the data size and will require adjustments to be made accordingly. Therefore, data processing and splitting strategies are necessary steps before modeling tasks.

### 3.2. Molecular Representation Methods for APIs and Excipients

When working with the dataset containing API and excipient molecular information in the original database, it is essential to convert the data into machine-readable formats. Molecular representation is a method to encode chemical identities based on chemical compositions and atomic configurations [54]. Three of the most important molecular representations, the International Chemical Identifier (InChI), the Simplified Molecular-Input Line-Entry System (SMILES), and the Molfile (MDL) have been incorporated with the Variational Autoencoder (VAE) algorithm to accurately generate molecular representation [55]. In addition, other representation methods including molecular descriptors and Extended-connectivity fingerprints (ECFPs) were also used in chemical reactions and formulation development [56,57]. Some open-source packages, such as RDKit, can be utilized to implement molecular representation [58].

## 4. Overview of AI Algorithms in Solid Dosage Forms Development

**Figure 2:**
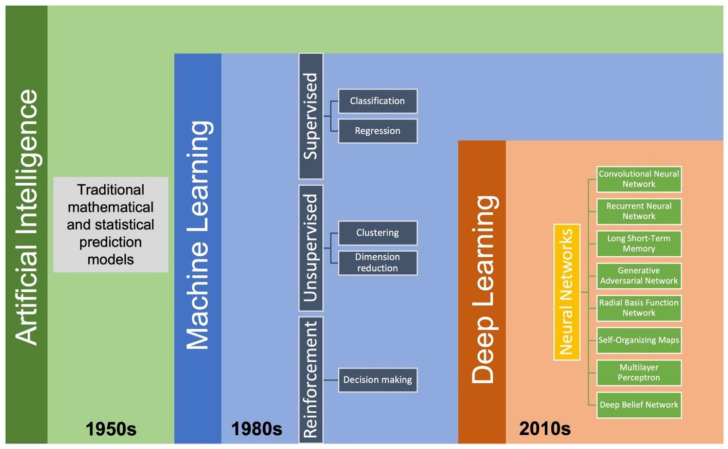
The development timeline for AI and its subfields.

Various AI-based models have successfully been applied in pharmaceutical solid dosage form development in recent years. Artificial intelligence is a combination of computer science, data analytics, and mathematics. As a subfield of AI, ML can typically be classified into supervised learning, unsupervised learning, and reinforcement learning (Figure 2). Supervised learning is a type of algorithm that consists of output/target variables that will be predicted from a set of input variables. A function of the input vs. desired output will be generated during the training process and will achieve the desired level of accuracy. Several supervised learning algorithms such as linear regression, logistic regression, decision tree, K-Nearest Neighbors (KNN), Random Forest, XGBoost, LightGBM, and Support Vector Machine have been extensively used for developing solid dosage formulations [56,59,60,61]. Unsupervised learning is an algorithm comprised of clustering and feature-finding methods which manages only the input variables. Reinforcement learning is primarily driven by specific decisions in a given environment where the computer will get either rewards or penalties for the actions it performs so that the model trains itself to achieve maximum performance [62]. Deep learning (DL) is a subfield of ML which includes state-of-the-art algorithms such as artificial neural networks and learns from a large amount of experimental data. Deep learning algorithms are built to accomplish prediction by introducing a highly sophisticated structure of models. In DL models, data is typically transformed through neurons of multi-layered neural networks in a non-linear approach [63]. In recent years, some DL algorithms, such as convolutional neural networks and recurrent neural networks, have been successfully introduced into the pharmaceutical sciences for different purposes when developing solid dosage formulations such as detecting tablet defects [64,65], predicting storage stability [59], predicting particle flowability [66], and predicting drug dissolution profiles [67]. To execute the AI modeling process, algorithms are written in programming languages such as Python, Lisp, C++, JavaScript, Java, and Haskell [68]. In addition, several commercially available software and platforms, including Google Cloud Machine Learning Engine, Azure Machine Learning Studio, TensorFlow, Cortana, and IBM Watson, can be used to implement different ML tasks [69].

### Advantages and Disadvantages of Different Algorithms

After completing data mining and processing, it is necessary to identify an optimal ML algorithm for the modeling process. More importantly, various factors of the database (such as dimensionality, size, and complexity) and factors of the modeling processes (such as training cost and time and inference time) need to be considered. Therefore, we summarized the advantages and disadvantages of some representative algorithms in Table 2 below. Table 2 provides general guidance for selecting suitable algorithms in the earlier stages of model development. For example, linear regression is a representative regression algorithm, an analysis using independent variables to predict dependent variables. Cost function and gradient descent methods help to optimize the model by minimizing the error. Random Forrest (RF) is one of the most widely used classification models. As an ensemble model, RF consists of a large number of decision trees used for prediction. More importantly, combining large amounts of uncorrelated trees outperforms individual trees by preventing errors. K-means clustering is an important unsupervised learning algorithm that involves aggregating numerous data points based on their specific similarities. The training process of K-means clustering starts with a group of randomly selected centroids for clusters and then optimizes the positions of the centroids by iteration. The ANN is one of the pharmaceutical industry’s most popular deep learning algorithms [34]. An ANN consists of the input, hidden, and output layers. A specific number of neurons exist in each layer. The neurons biologically simulate the human brain and are used to transfer signals in an ANN. A Convolutional Neural Network (CNN) is another popular deep learning algorithm that is more widely used for image analysis. A conventional CNN contains convolutional, pooling, flatten, and hidden layers. CNNs have been widely applied for image classification and segmentation tasks with the improvement of computational hardware such as GPU and CPU [64,70].

## 5. Model Predictive Performance Evaluation and Explainability

After the machine learning modeling process has been completed, it is necessary to evaluate the predictive performance of the models. Several different metrics can be used to assess the predictive performance, and we can divide them into regression and classification metrics. Table 3 is a summary of the different metrics in different applications. For regression modeling tasks, coefficient of determination (R^2^), mean squared error (MSE), root mean squared error (RMSE), and mean absolute error (MAE) are typically used as metrics for evaluation. For classification modeling tasks, a confusion matrix will first be applied to calculate some metrics, including accuracy, precision, recall, F1-score, sensitivity, and specificity. However, the results from some classification metrics, such as accuracy, are misleading in the imbalanced classification modeling task. Therefore, additional evaluation metrics such as Receiving Operating Characteristic (ROC) area under the curve (AUC) and Cohen’s Kappa are employed for model evaluation with an imbalanced dataset [71]. In recent years, deep learning-based image analysis, a subfield of machine learning, has been introduced for developing solid dosage formulations with its use in the pharmaceutical field continuing to expand rapidly [64,72]. Unlike machine learning modeling for tabular data, deep learning-based image analysis evaluation metrics are based on calculating pixels or voxels in the images. Therefore, other metrics such as Average Precision (AP) and mean Average Precision (mAP) are typically used for object detection tasks [73]. Pixel accuracy, Intersection-Over-Union, and Dice Coefficients are used for image segmentation tasks [74].

**Figure 3:**
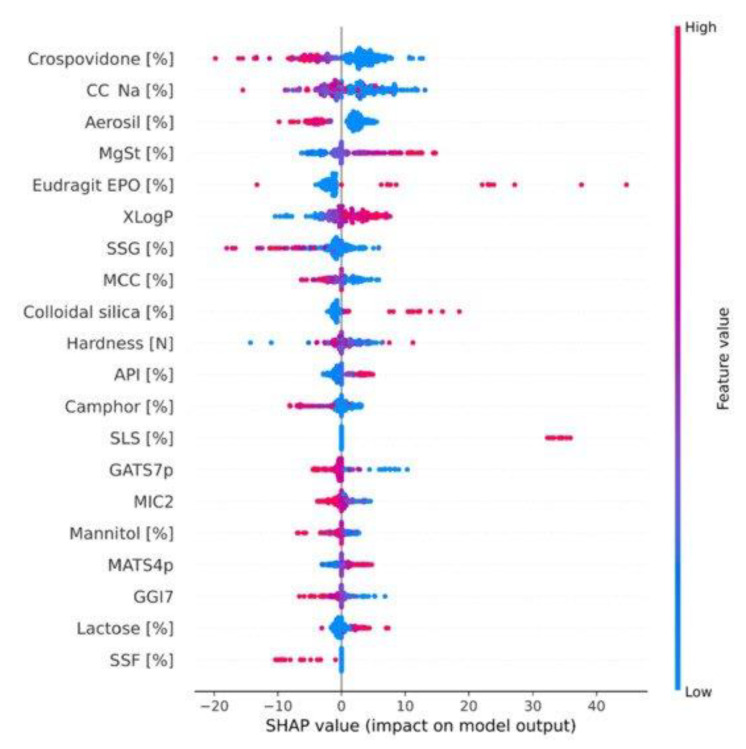
SHAP dependence plot of the top 20 features of the deep learning model. MCC, microcrystalline cellulose; CC-Na, croscarmellose sodium; SSG, sodium starch glycollate; MgSt, magnesium stearate; SSF, sodium stearyl fumarate; API, active pharmaceutical ingredient. The color bar depicts the feature values, and the dots’ *X*-axis position exhibits their correlation with the disintegration time. (Adapted with permission [79]. 2022, Pharmaceutics).

**Figure 4:**
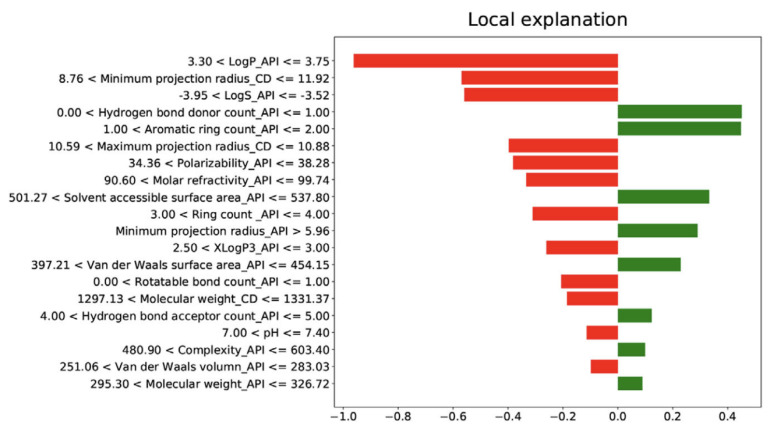
LIME interpretation results of cyclodextrin (CD) formulation. LogP_API, LogP of API; Minimum projection radius_CD, Minimum projection radius of CD molecule; LogS_API (Adapted with permission [61]. 2021, Food Frontiers).

Feature importance and model explainability are essential steps after the model evaluation. Feature importance is indicated by the scores of all variables in the model used for prediction—the higher the scores, the more significant the effects of that specific variable in the model. Several feature importance methods, including F-statistics, impurity reduction, permutation importance, absolute importance, main factor, and maximal information coefficients, were widely used to explain the trained models [75,76]. More importantly, to better understand the feature importance from the sample level and to identify if each prediction is trustworthy, some advanced model-explainability techniques such as Shapley Additive exPlanations (SHAP) and Local Interpretable Model-agnostic Explanations (LIME) have been used for implementation [77,78]. For example, Szlęk et al. successfully applied SHAP in a machine-learning model of orally disintegrating tablets [79]. In this study, a deep neural network was trained for the prediction of the disintegration time of tablets and achieved an R^2^ of 0.84. To evaluate the model explainability, SHAP values of 39 input variables were computed and analyzed (Figure 3). Based on the SHAP results, higher disintegration times are typically attributed to a higher amount of disintegrants and fillers. However, an opposite correlation was observed for the lactose amount. In addition, Ye et al. implemented the LIME method for the post hoc interpretation of LightGBM-based cyclodextrin (CD) formulation model [61]. According to the LIME analysis results, the LogP of API, the minimum projection radius of CD, the LogS, the hydrogen bond donor count, and the aromatic ring count of API can jointly make the prediction of the binding-free energy when forming a CD complex (Figure 4).

## 6. Applications of AI in Solid Dosage Forms

### 6.1. Overview of Solid Dosage Formulations Designed by AI

Solid dosage forms including tablets, powders, and granules remain the most widely used form of drug products because of their ease of use and patient compliance. In all likelihood, these products will continue to dominate the pharmaceutical market in the future. Researchers and scientists began studying AI applications in solid dosage forms in the 1990s. According to the published literature, the number of related publications on AI in solid dosage forms has increased annually by 100% since 2015 [34]. Among all solid dosage forms, tablets attract people’s attention the most and represent over 60% of AI-related solid dosage form development [34]. To better understand how AI algorithms can be applied to different solid dosage formulations, we summarized the recent AI applications to provide a holistic picture of this research area (Table 4).

### 6.2. Tablets

A tablet is one of the most essential oral solid dosage forms. A tablet consists of a mixture of APIs and excipients and is typically prepared by compression or molding [96]. The function of excipients used for preparing tablets can be classified as (1) enhancing tableting performance: lubricants, glidants, binders, and diluents, (2) masking taste and improving appearance: sweeteners and food pigments, and (3) modifying drug release: disintegrants and sustained-release polymer coating [97]. In this section, several applications of AI in tablet formulations including predicting drug release, optimizing critical processing attributes during the manufacturing process, and detecting tablet defects will be discussed.

#### 6.2.1. Predicting Drug Release

Drug release studies, including in vitro and in vivo, are two of the most fundamental pre-clinical experiments conducted during product development. The drug release profiles are affected by critical material attributes and critical processing parameters. For example, minimal changes in the compaction parameters, such as pressure and tablet geometry, or other variables such as drug loading, may significantly influence dissolution rates. In addition, a conventional in vitro drug release study requires specific equipment such as apparatuses, UV-Visible spectrophotometers, and USP-approved vessels. The entire analysis process of these conventional studies is time-consuming. With assistance from AI technology, scientists can now predict important characteristics of drug formulations and therefore improve the product development process by saving time and cost. The balance of this section will describe three published studies that used AI to predict dissolution profiles, drug release profiles, and disintegrating times of various types of tablets.

In a 2021 publication, Galata et al., investigated the prediction of dissolution profiles of hydrophilic matrix sustained-release tablets by applying three AI algorithms. In this study, ANN, Ensemble of Regression Trees, and SVM were used for the data analysis and dissolution profile prediction. In addition, critical Material Attributes (CMAs) and Process Analytical Technology (PAT) results were combined as input data to obtain a database for modeling. The results indicated that Particle Size Distribution (PSD) is one of the most significant variables for the model prediction. Furthermore, ANN was identified as the most accurate model among all models in the evaluation metrics [80].

In another study published in 2012, the prediction of drug release in matrix tablets was evaluated using an *Elman* dynamic neural network, decision trees, and multilayer perceptrons. Different types of tablet matrixes containing polyethylene oxide polymer or glyceryl palmitostearate were formulated under various compression forces. The input variables included the CMAs and other tablet properties such as tensile strength and porosity. *Monte Carlo* was used as an optimizer for neural networks and difference (f1) and similarity (f2) were calculated to evaluate the accuracy of the models. The results showed that as a subfield of RNN, the *Elman* dynamic neural network performed the best and exhibited precise prediction on drug release [98].

**Figure 5:**
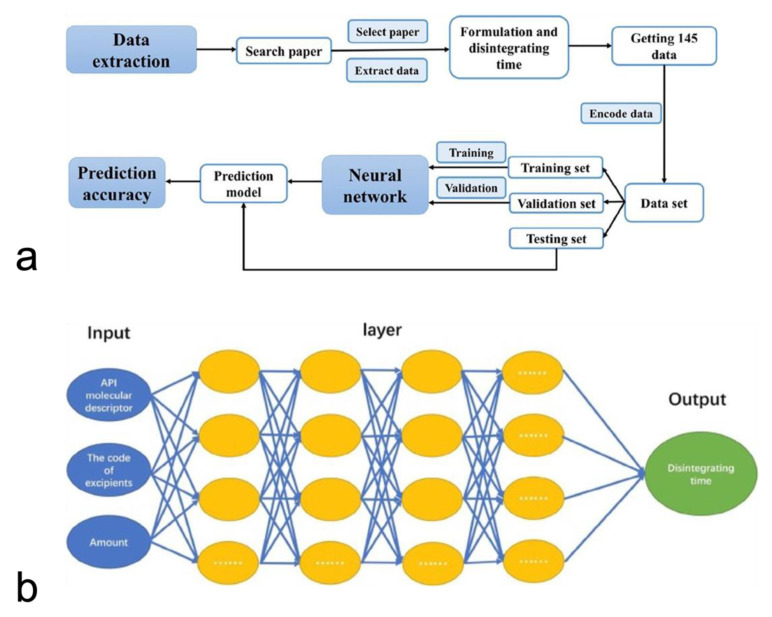
The workflow of AI-based tablet’s disintegrating time predictive model (**a**). The deep neural network’s structure (**b**) (Adapted with permission [100]. 2018, Asia Journal of Pharmaceutical Sciences).

Finally, Han et al. studied the application of deep neural networks in predicting the disintegrating time of tablets [99]. In this study, data on 145 drug formulations were collected by literature mining and then split into training and testing subsets using the improved maximum dissimilarity algorithm (MD-FIS). Notably, MD-FIS is an advanced data selection method, and it enables the testing and validation data sets to be representative (Figure 5). A deep neural network consisting of ten hidden layers with 50 neurons in each layer was built up for the modeling process (Figure 5). The fine-tuned deep neural network achieved high accuracies in both validation and testing sets of 85% and 80%, respectively. Therefore, ML models have been successfully applied to predict the drug release profiles of tablets.

#### 6.2.2. Developing 3D-Printed Tablets Using AI

Three-dimensional (3D) printing is one of the most innovative techniques for personalized medicine with the potential to produce tablets considering the physiology, genetic profiles, and drug response of patients [101]. Several methods have been used to prepare personalized 3D-printed tablets such as fused filament fabrication, binder jetting, selective laser sintering, pressure-assisted microsyringe, and stereolithography [101]. During the manufacturing process, parameters such as nozzle temperature, platform temperature, and printing speed play a crucial role in controlling the quality of the final products, and these parameters may also affect the drugs’ in vitro and in vivo release profiles [100]. Therefore, to optimize the 3D printing process and reduce the experimental workload with numerous variables, AI technologies show great potential to be incorporated into this technique and identify the design window.

**Figure 6:**
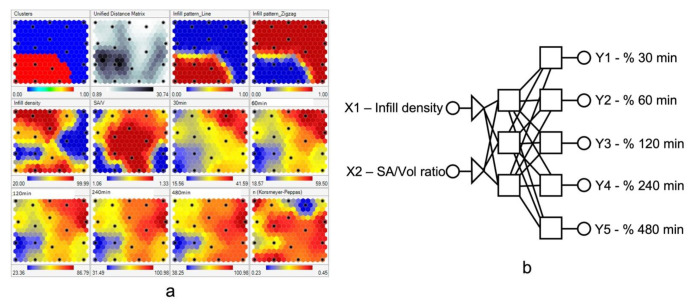
(**a**) Self-organizing maps of drug release profiles. (**b**) ANN structures. The size, number, and position of each unit on the maplet surfaces provides information on the data distribution. Each black dot on the same position of multiple maplet surfaces represents the same formulation. Therefore, the correlation between different variables can be interpreted by observing the units at the same positions on different maplet surfaces. (Adapted with permission [82]).

The following studies have demonstrated the applications of AI in optimizing processing parameters during the 3D printing process. Obeid et al. studied the effects of processing parameters and tablet surface area/volume ratio on diazepam 3D-printed tablets’ drug release using the ANN model. In this study, processing parameters, including an infill density ranging from 20% to 100% and an infill pattern, were used as input variables, and the dissolution rate was set as a target. First, self-organizing maps (SOM) were applied to visualize and interpret the interaction between different variables (Figure 6a). After SOM analysis, infill density and surface area/volume ratio (SA/V) were selected as input variables for further modeling studies. Then, a three-layer ANN containing (1) two neurons in the first layer, (2) three hidden neurons in the second layer, and (3) five neurons in the third layer was built for modeling (Figure 6b). After the ANN modeling and validation, high dissolution rates were achieved under conditions of lower infill density (<50%) and a zigzag infill pattern. Most importantly, the dissolution rates of diazepam tablets were precisely predicted by the ANN model [82].

In another recent study, processing temperature, printability, and feedstock characteristics of 3D printed tablets were successfully predicted by applying multiple ML models (i.e., RF, SVM, and ANN) from 968 formulations in published literature. The first step in preparing the 3D-printing tablets by fused deposition modeling (FDM) technology is to obtain filaments using hot melt extrusion. As a critical processing parameter, extrusion temperature is vital in controlling the filament’s properties including diameter, strength, and texture. Therefore, it is necessary to optimize the extrusion temperature to maintain product quality. In this study, ANN was found to accurately predict the extrusion temperature with an R^2^ of 0.90 and a mean absolute error (MAE) of 5.18 °C. In addition, the printing temperature, a processing parameter used to determine printability, could be predicted by the RF algorithm with an R^2^ of 0.86 and a mean absolute error (MAE) of 6.87 °C [71].

More importantly, deep learning has demonstrated great potential in 3D printed tablet defect detection by providing a computer-aided, non-destructive quality assurance method. Westphal et al. investigated the feasibility of detecting defects in tablets prepared by selective laser sintering 3D printing technology by using CNN [65]. Specifically, multiple CNN pre-trained models, including VGG16 and Xception were applied for this image classification task. The results showed that VGG16 exhibited the highest accuracy of 95.8%. In addition, Grad-CAM was successfully applied for the CNN model visualization and explanation, and VGG16 showed a more precise localization of the effects than Xception [65].

#### 6.2.3. Detecting Tablet Defects

Tablet defects such as cracking, capping, binding, and sticking are common behaviors during the manufacturing process. These defective tablets typically need to be screened out manually which requires a massive number of laborers with the process being a challenge to scale up. To address this problem, some techniques, such as X-ray computed tomography (XRCT), can be used to analyze the internal structure of tablets [64,102]. To expand the application of this technique, researchers have combined XRCT with the deep learning technique to successfully detect tablet defects [64].

**Figure 7:**
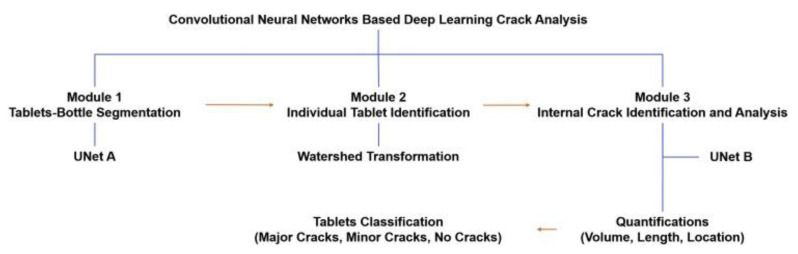
The workflow of the CNN-based deep learning method for detecting tablet cracks (Adapted with permission [64]).

Ma et al. studied the application of convolutional neural networks in detecting internal tablet defects. In this study, different batches of tablets containing excipients, including mannitol and microcrystalline cellulose, were prepared and then captured by XRCT for image analysis. An image augmentation strategy was employed, resulting in an increase of images from 573 to 43,548. A CNN containing the following three modules was used for the image analysis: (1) UNet A, which is used to distinguish the tablets from the bottle, (2) Module 2, which is an automated analysis used to identify individual tablets, and (3) UNet B, which can determine internal cracks of tablets quantitatively (Figure 7). During the model testing, The UNet neural network exhibited an accuracy of up to 94% for seven batches of tablets. In addition, this CNN method can potentially support the detection of defects from other products and may significantly reduce time, workload, and financial costs [64].

### 6.3. Powders

Powders are one of the most conventional and oldest pharmaceutical dosage forms. They consist of a dry substance composed of finely divided particles. Powders are the basis of many other dosage forms including capsules and tablets [103]. Pharmaceutical powders can be prepared by grinding, crushing, or comminuting, and they typically have particle sizes between 10 nm and 1000 µm [104]. AI technologies have been successfully applied to the process control of powder engineering for both small molecules and biologics. In addition, some studies have demonstrated the great potential of AI applications in carrier-based dry powder inhalation [87,88].

#### 6.3.1. Applications of AI in Process Control during Powder Engineering

The powder engineering technique involves using micronization or other methods to obtain particles with optimal particle sizes for different administrations including oral solid dosage forms or pulmonary delivery [105]. Particle size is an important indicator during pharmaceutical product development because it influences product properties and qualities such as surface area, solubility, porosity, bioavailability, powder flowability, and shelf life. For example, aerodynamic particle size is crucial for pulmonary drug delivery because the powders will be exhaled if they are too small (<1 µm) or cannot reach the lung if they are too large (>5 µm) [106]. To achieve the desired powders, several techniques including jet-milling, spray drying, supercritical-fluid, co-crystallization, and wet-polishing can be used to prepare the powders using the optimally-sized particles [107,108,109,110]. More importantly, critical processing parameters such as drying temperature, pressure, air flow, and energy input, play important roles in contributing to the quality or critical properties of the final product. Recently, some studies have demonstrated the feasibility of applying AI technologies for controlling product quality or critical properties during the particle engineering process.

Using machine learning tools, Chauhan studied the effects of different drying methods, such as spray-drying (SD) and freeze-drying (FD), on peptide stability and bioactivity. In this study, the rice natural peptide network (NPN) was first processed through both SD and FD; then, an ANN model was used to predict peptide bioactivity including anti-inflammatory activity. The results showed that the estimators exhibited an accuracy of up to 85% when predicting anti-inflammatory activity and suggested no significant difference was found between the different drying methods [86]. In addition, to further understand the drying kinetics, Keskes et al., applied AI models based on SVM and ANN and analyzed the effect of some critical attributes including initial mass, drying temperature, water content, and drying pressure on the drying time. The AI models exhibited precise prediction with an R^2^ of 0.999 and root-mean-square error RMSE less than 8.810405 × 10^−3^ [111].

In another study, a multilayer perceptron ANN was applied to predict exergetic performance during the SD process. Processing parameters including drying air temperature, aspirator rate, spray air flow rate, and peristaltic pump rate were used as input variables. Exergetic performance, which was described by parameters such as inlet exergy, outlet exergy, entropy generation, and exergy efficiency, was treated as the output. The results showed an R^2^ of 0.98 for the ANN model, demonstrating that AI could achieve excellent performance-predicting exergy efficacy during the SD process [112].

#### 6.3.2. Applications of AI in Designing Dry Powder for Inhalation

Dry powder is one of the most widely used dosage forms to deliver drug formulations into human lungs, and a capsule-based dry powder inhaler is a preferred device for patients [113]. Aerosol performance is an essential indicator for the product development of dry powder for inhalation that needs to be controlled carefully. The aerosol performance of dry powders can be determined by fine particle fraction (FPF), median mass aerodynamic diameter (MMAD), and geometric standard deviation (GSD) using instruments such as a next-generation impactor or a cascade impactor [114]. Using AI tools, these parameters can be predicted by modeling which plays a crucial role in dry powder inhalation product development.

**Figure 8:**
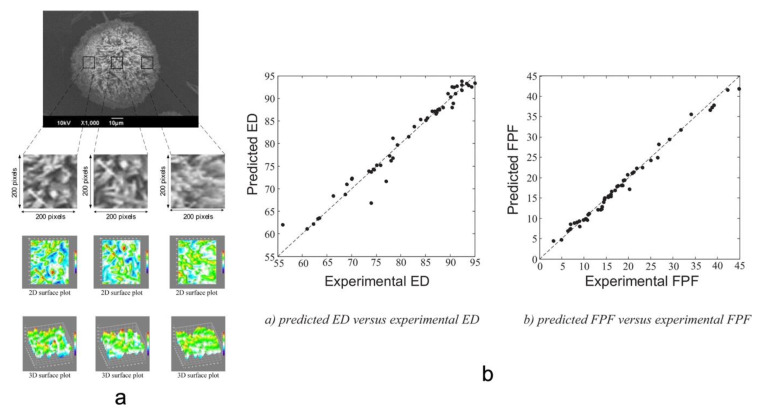
(**a**) The workflow of processing and quantifying SEM images using ImageJ. (**b**) Parity plots of experimental ED and FPF vs. predicted ones (Adapted with permission [87]).

Farizhanidi et al. studied a machine learning approach for designing a carrier-based dry powder for inhalation. Sixty-five datasets containing three carriers and three drugs were used for analysis. The input variables of the database consisted of (1) CMAs, and (2) quantitative variables such as root mean square deviation (Rq), skewness of the assessed profile (Rsk), and mean polar facet orientation (FPO), all from scanning electron microscopy (SEM) images (Figure 8a). Fine particle fraction and emitted dose (ED) were used as the output. A feedforward ANN model was built up for modeling, and the database was divided into 50 subsets for training and 15 subsets for testing. The model showed an accuracy with an R^2^ of 0.9820 and 0.9556 for FPF and ED (Figure 8b), respectively, and showed significant improvement over empirical modeling. This study demonstrated the feasibility of designing dry powder inhalation products using AI technologies [87].

### 6.4. Capsules

Capsules are drugs enclosed in a shell made from gelatin or other materials. Capsules are another one of the solid dosage forms most widely used, a particularly for oral administration. However, limited literature exists describing the application of AI methods in developing capsule-based formulations. To obtain different drug release profiles, several types of capsules including hard gelatin, soft gelatin, modified release, and enteric capsules have been used to encapsulate the drug powders. Zhou et al. demonstrated the feasibility of identifying capsule defects using an enhanced CNN [92]. In this study, capsules with different defects including holes, concave heads, uncut bodies, and oil stains, as well as capsules that were shriveled, locked, or nested, were first prepared manually. The enhanced CNN was equipped with L2 regularization and an Adam optimizer which were used to overcome the overfitting of the model. In addition, K-Nearest Neighbor (KNN) and Support Vector Machines (SVM) were also employed in this study for comparison. The results from the confusion matrix showed an accuracy of up to 97.56% for detecting capsule defects when applying this enhanced CNN model [92].

### 6.5. Granules

Granules are another pharmaceutical solid dosage formulation that consists of aggregates of powder particles with drugs and excipients. Granules offer the advantage of flexible administration for patients who have difficulty swallowing capsules or tablets and are more shelf-stable than liquid forms. Capsules also offer improved flowability and compressibility compared to drug bulk powder. This dosage form can be classified into several types: modified-release granules, coated granules, effervescent granules, and gastro-resistant granules. Wet granulation and dry granulation are the two main methods used to prepare granules [115]. Recently, some studies have shown applications of AI tools in manufacturing granules, in the areas of process control and the prediction of final particle size [60,94].

Mariana Landin investigated optimal impeller power during high-shear wet granulation processing using an AI tool containing neuro-fuzzy logic and gene expression programming. In this study, input variables including volume, impeller diameter, impeller speed, liquid ratio, wet mass density, and mean torque were fed in for the modeling process. The prediction results indicated a high correlation (R^2^ > 86.78%) for different batches ranging from 25 L to 600 L. In addition, the results have shown the great potential of estimating the endpoint of the high-shear granulation process by predicting the final impeller power [94].

Zhao et al. studied the evaluation and prediction of drug contents in sugar-free granules by using AI techniques. In this study, near-infrared (NIR) spectroscopy first demonstrated the feasibility of measuring drug contents in granules. Subsequently, different machine learning methods were applied to predict the drug remaining based on the NIR spectrums. Finally, three AI approaches were optimized for modeling development: backpropagation ANN, particle swarm optimization SVM, and a genetic algorithm. The results demonstrated that AI models are suitable tools for the quantification of the drug content in granules [116].

In another study, the particle size distribution of the final granules was modeled by different AI tools including ANN, genetic programming, and multiple linear regression. The granules containing microcrystalline cellulose, lactose, and mannitol were first prepared by using an oscillating mill. Then, material properties such as true density and process parameters, including impeller tip speed and compaction force, were used for analysis and modeling. Based on the evaluation metrics results of the three AI models, ANN achieved the best prediction performance with a normalized mean squared error (NRMSE) of 2.28% and R^2^ = 0.9926 [60].

### 6.6. Solid Dispersions

As one of the most important solubilization methods for solid dosage forms, solid dispersions are typically composed of drugs and polymers. Amorphous solid dispersion is a subfield of solid dispersion. Amorous solid dispersion occurs when an amorphous drug is molecularly dispersed into a polymer matrix, also known as a homogeneous drug-polymer solution [117]. A solid dispersion can be obtained by (1) a fusion-based method such as hot-melt extrusion, and (2) a solvent-based method such as spray-drying, and co-precipitation [118]. However, due to environmental factors such as heat, moisture, storage time, and drug-polymer interaction, solid dispersion is physically and chemically unstable which results in drug-polymer phase separation over time [117]. Therefore, it is crucial to consider the critical factors including stability, miscibility, and solubility, to overcome phase separation happening during the product development of solid dispersions. A conventional development pathway for solid dispersion consists of the pre-formulation, formulation, and characterization stages which are time-consuming and laborious, even though some high throughput screening methods such as solvent casting can be applied [119]. To improve the efficiency and mitigate relatively high labor intensity during the development of solid dispersion products, some AI-based techniques were successfully employed to predict some characteristics including physical or chemical stability, dissolution rate, and dissolution profile of solid dispersion formulations.

#### 6.6.1. Predicting Physical or Chemical Stability

Han et al. studied the feasibility of predicting the physical stability of solid dispersion by using several machine learning methods including ANN, SVM, RF, LightGBM, KNN, and naïve Bayes [59]. In this study, 50 drug molecules containing 646 physical stability data points were collected from the public database for training the model. Molecular descriptors such as molecular weight, melting point, hydrogen bond acceptor count, and heavy atom count were used as molecular representations to generate the database. In addition, an accelerated stability study was conducted for three months and six months under 40 °C/75 RH to evaluate the model’s performance in predicting its physical stability. The results revealed that RF was the best predictive model among others, with an overall accuracy of up to 82%. Then, a 17β-estradiol (ED)-polyvinylpyrrolidone (PVP K30) solid dispersion was prepared by solvent evaporation for experimental validation. The validation experimental results showed that a 1:5 ratio solid dispersion system was stable for 6-months under accelerated conditions, while a 1:2 ratio solid dispersion system was unstable, which corresponded to the ML predictions. This is because drug loading played an important role in physical stability by affecting the number of hydrogen bonds between the drug and excipient and steric hindrance of the solid dispersion system [59]. In addition, chemical stability and drug-excipient compatibility are other critical factors and must be considered during formulation development. Wang et al. successfully developed PharmDE as an integrated platform for drug-excipient incompatibility risk prediction by analyzing 532 data points from 228 published articles, which potentially facilitated the initial screening of solid dispersion [120]. In conclusion, AI technologies have been demonstrated to predict the physical and chemical stability of solid dispersion and can potentially accelerate the product development process by shortening the stability testing time and narrowing down the number of testing samples.

#### 6.6.2. Predicting Dissolution Rates and Profiles

For some solid dispersions, the dissolution profile can be summarized by the “spring and parachute” effect, where the “spring” represents the rapid dissolving and supersaturating of drugs, and the “parachute” describes precipitation led by the recrystallization of amorphous drugs [121]. In contrast, some solid dispersions can maintain the supersaturation with the addition of excipients and will not precipitate over time. Dong et al., developed a method based on AI to predict the dissolution types and the dissolution rate [56]. This study first used literature mining to obtain a database of 702 dissolution curves containing 50 APIs and 25 polymers. Then, various AI algorithms were employed for the modeling process, including RF, SVM, LightGBM, and XGBoost. The descriptors of the APIs and polymers were obtained through molecular computational software. The molecular descriptors were combined with processing conditions such as temperature, drug loading, and volume and were then used as input variables. The dissolution type was recognized as a binary output and could be either supersaturation or precipitation, while the dissolution rate was another output and a regression target. For dissolution type, ECFP4-XGBoost was found to have the highest accuracy of up to 97.7%. Random Forrest, SVM, and LightGBM achieved a high accuracy (R^2^ is between 0.809 to 0.928) when predicting the dissolution rate [56].

In addition, experimental validation of ML models is critical to further test the model’s prediction performance and real-life compatibility prospectively. Gao et al., systematically studied the applications of integrated computer-aided technologies to design a ternary solid dispersion [122]. In this study, a LightGBM model was first used to predict the inclusion’s binding free energy between the drug (andrographolide) and different types of cyclodextrins. Then, γ-cyclodextrin showed the strongest predicted binding affinity, which was also validated experimentally by a solubility study. Moreover, molecular dynamic simulation was utilized to understand the inclusion mechanism. Most importantly, cell and animal experiments were performed to validate the ML model, and the presence of D-α-Tocopherol polyethylene glycol succinate (TPGS) greatly increased the intracellular uptake in cells. The model showed an excellent overall performance in predicting the pharmacokinetic (PK) parameters (i.e., C_max_, T_max_, and AUC) in rats. The ternary system (andrographolide-CD-TPGS) demonstrated increased relative bioavailability of 2.6-fold and 1.59-fold compared with pure drug and commercial dropping pills, respectively [122].

### 6.7. AI Applications in Pharmaceutical Image Analysis

In pharmaceutical science, especially in solid dosage formulations, visual inspection is one of the most important methods for the characterization and quantification of APIs, excipients, and dosage forms [123]. US Pharmacopeia (USP) has specified the visual inspection for injections which requires that the final products do not contain sub-visible particles. Farkas et al. summarized this type of image analysis into static and dynamic image analysis [123]. Static image analysis is a method in which the particles are motionless during image acquisition. The images can be captured by techniques such as bright field microscopy, confocal laser scanning microscopy, fluorescent microscopy, microfocus X-ray imaging, scanning electron microscopy, and polarized light microscopy [123]. Dynamic image analysis is another method with repeatable results which involves some techniques such as process analytical technology (PAT), in-line photometric stereo imaging, and dynamic foam analyzer [123]. Deep learning has gained more attraction in medical image analysis for several years and has been widely used for image classification, object detection, image segmentation, registration, and other tasks [124]. In addition, the deep learning-based image analysis method has recently been applied in pharmaceutical science and shows great potential in some fields. Specifically, three published studies of applications of AI in predicting in-vitro drug release, measuring tablet disintegration rate, and analyzing particle size will be described in the remainder of this section. We believe deep learning-based image analysis provides significant benefits such as high accuracy, high efficiency, reduced workload, and high adaptability during solid dosage formulation development.

#### 6.7.1. Image Pre-Processing Methods

Deep learning-based image analysis has gained more attention in the pharmaceutical industry as it is crucial to ensure image quality before feeding images into the models. Therefore, to obtain a robust model, it is necessary to perform some image pre-processing techniques such as resizing, normalization, contrast adjustment, and image augmentation [125]. Image batches should be uniform in the same heights and widths before being fed into the model. In addition, the aspect ratio, which is the ratio of the width and length of the images, should be constant for all images. Image contrast and brightness are other important factors that must be considered for image pre-processing. Normalization is defined as the rescaling of the pixel values to 0–1, resulting in a faster model training process. Some images show relatively low intensity and contrast during experiments, which makes these images challenging for the deep-learning model to analyze. To address these issues, some image pre-processing techniques such as Contrast Limited Histogram Equalization (CLAHE) have been successfully applied to improve the images’ contrast, resulting in improved model performance [126]. Image augmentation is a process for enlarging an image dataset by applying techniques such as horizontal and vertical flip, shift, rotation, and transposing to improve the robustness of the model and potentially prevent overfitting [127]. In summary, image pre-processing techniques are important for AI-based image analytics.

#### 6.7.2. Case Studies of AI-Based Image Analysis

Xi et al. successfully developed a method for measuring the particle size distribution of spray-dried particles using XRCT images [89]. This study used an AI-facilitated tool to quantitatively study thousands of individual particles. These image analysis results have demonstrated a high correlation with the measurement by laser diffraction. More importantly, this method can potentially facilitate the development of spray-dried particles with optimized performance [89].

In another study by Liu et al., deep learning-based XRCT image analysis was successfully applied to visualize and estimate the drug release rate of long-acting parenteral implants [72]. Specifically, image segmentation and analytics were utilized to visualize the drug distribution with the implants and to facilitate an understanding of the quantitative structural information. The drug release prediction based on the voxel calculation from XRCT images showed relatively poor performance with about a two-fold over-prediction. With the addition of FIB-SEM (focused ion beam scanning electron microscopy), a good agreement on the in-vitro drug release between the experimental and the prediction results were achieved with about a 10% and a 5% difference for low drug loading and medium drug loading implants, respectively. The potential of image analysis for understanding the drug release mechanism and the microstructure of implant development has been demonstrated in this study [72].

Disintegration testing is one of the most critical steps for the product development of immediate-release tablets. However, the current guidance by the USP of <701> only provides information on the duration of the disintegration process, which is variable, subjective, and prone to human error. Recently, scientists have successfully developed a Computer Vision for Disintegration (CVD) system for the detection of and the quantitative measurement of the tablet disintegration rate with an accuracy of up to 99.6% [84]. Briefly, the tablet disintegration images were captured by a camera, and then a CNN was utilized to analyze and interpret the data. This technology platform allows for a more extensive understanding of the tablet disintegration process beyond merely analyzing the duration [84].

Finally, UV and Vis PAT image technology was integrated with machine learning modeling to analyze particle size distribution in tablets [85]. In this study, UV and Vis imaging-based machine vision systems coupled with pattern recognition neural networks were built up for particle size classification. As a result, the fine-tuned deep learning model achieved a high accuracy of 97% when analyzing the particle size distribution of meloxicam tablets. Most importantly, this method can provide a rapid, non-destructive, and in-line tool for particle size analysis of tablets [85].

## 7. Prospects

Even though AI-based models have been widely introduced in formulation development, there are some areas that have not yet been discovered that are worth exploring. For example, advanced deep learning algorithms such as graph convolutional networks (GCN) and generative adversarial networks (GAN) have been widely used in chemistry and material science. Kojima et al. studied the application of GCN in predicting the compound-protein interaction, and the model provided the visualization of atomic contributions to the prediction [128]. In addition, GAN becomes more critical in drug discovery because it facilitates exploring and optimizing the chemical design space for the desired functionality [129]. However, there is limited literature on the applications of GCN and GAN in drug formulation development and this area can be explored in the future. Moreover, some applications of AI/ML in solid dosage development processes have yet to be investigated. For example, deep learning-based image analysis technology can extract a variety of properties of the formulations including particle size distribution [89]. It would be significant if the image analysis were incorporated with a process analytical technology (PAT) instrument for the in-silico measurement of some properties during the manufacturing process. Finally, most of the methods in the published literature reflect supervised learning, and reinforcement learning is a robust learning algorithm yet to be studied.

## 8. Conclusions

In summary, we have highlighted how AI tools can be harnessed for developing solid dosage formulations and how they can be utilized in different formulations. Though most studies have demonstrated that AI can revolutionize the drug discovery pipeline, AI has also shown more and more potential in formulation development. Compared to the conventional formulation development pathway which uses the trial-and-error method and requires a laborious workload, an AI-based development strategy tends to expedite the development process by allowing scientists to generate low-cost predictions in a relatively rapid manner. This review also discussed multiple AI algorithms used for various tasks and provided general guidance for model selection to better help scientists to integrate AI techniques into their research. In addition, we introduced data processing and model evaluation strategies to provide a tool for systematically understanding and implementing AI models. More importantly, we categorized different pharmaceutical formulations and summarized their available AI prediction models in the published literature.

In recent years, the deep learning-based image analysis method has attracted increasing attention and has become a practical approach for the critical properties’ prediction of drug formulations. However, the current AI modeling methods in solid dosage formulation have limitations. For example, obtaining a large balanced database is still relatively difficult, and published articles tend to be biased toward positive results. Additionally, most current studies performed retrospective experimental validation, while only a few conducted experimental validations prospectively. Therefore, we believe expanding the utility of AI in pharmaceutical solid dosage formulations offers both a challenge and an opportunity for the pharmaceutical research community.

## Figures and Tables

**Table 1:** Some commonly used databases containing information on solid dosage formulations.

Some Popular Databases of Solid Dosage Formulations				
	Name	Size	Publisher	Reference
APIs/Chemicals	US Pharmacopoeia	>5000	US Pharmacopoeia Convention	[36]
	PubChem	>111 million	National Center for Biotechnology Information (NCBI)	[37]
	Cambridge Structure Database	>900,000	University of Cambridge	[38]
	SciFinder	142 million	Chemical Abstracts Service (CAS)	[39]
	Merck Index	>10,000	Royal Society of Chemistry (RSC)	[40]
Excipients	Inactive Ingredient Search for Approved Drug Products	9438	U.S. FDA	[41]
Formulations	Drugs@FDA (FDA-Approved Drugs)	>20,000	U.S. FDA	[42]
	Orange Book (Approved Drug Products with Therapeutic Equivalence Evaluations)	N/A	U.S. FDA	[43]
	DrugBank	>500,000	University of Alberta	[44]
	Dissolution Methods	1388	U.S. FDA	[45]
	MedlinePlus^®^	∼1500	National Institute of Health	[46]
	Drug Information Portal	>49,000	National Institute of Health	[47]

**Table 2:** Summary of the advantages and disadvantages of different AI algorithms.

Advantages and Disadvantages of Different AI Algorithms			
	Algorithms	Advantages	Disadvantages
Regression	Linear regression	Easy to implement Efficient to train Performs well for linearly separable data	Prone to overfitting and noise The assumption of linearity of dependent and independent variables
	Lasso regression	Performs shrinkage and variable selection Good prediction and interpretation	Model selection is unstable
	Ridge regression	Can avoid overfitting Performs well when having high-dimension data Does not require unbiased estimators	Unable to perform feature selection Shrinks the coefficient towards 0 Trades off bias for variance
Classification	K-Nearest Neighbors	No training periods Easy to implement	Sensitive to missing values and outliers Does not work well for high-dimensional data Poor performance when having large databases
	Support Vector Machines	Performs well when classes are separable Performs well in higher dimensions Outliers are less impactful	Slow processing speed Poor performance when having overlapped classes Challenging to select appropriate hyperparameters
	Random Forrest	Good performance when having imbalanced data Minimizes errors Can deal with massive databases Good handling of missing data Less impact of outliers	Easier for overfitting Relatively low accuracy Black box algorithm
	Naïve Bayes	Scalable databases Real-time and fast predictions Compatible with high-dimensional data	Poor performance of the estimator The assumption that variables are independent is not always true
Clustering	K-means clustering	Easy to implement Fast computation time with huge variables Can recover from failure automatically	Sensitive to noisy data and outliers Need to specify the number of clusters (k) in advance
	Density-Based Spatial Clustering of Applications with Noise clustering	Does not require specification of the number of clusters in advance Performs well with arbitrary shaped clusters Robust to outliers	Poor performance when data has high dimensions Fails when having varying cluster density
	Mean shift clustering	It can be used for complex clusters Robust to outliers It only needs bandwidth to determine the number of clusters	Poor performance when having high-dimensional data Slow implementation time
Deep learning	ANN	Can store information on the entire network Exhibits fault tolerance Has distributed memory Gradual corruption Can perform multi-tasking simultaneously	A relatively high requirement in terms of hardware Poor explainability Challenging to determine ANN structure Unknown duration of the networks
	CNN	High accuracy once CNN is fine-tuned Can detect the important features or patterns in the images	Requires higher computational power, especially GPU Large training data required
	RNN	Can model a collection of records The assumption that each pattern is dependent on the previous ones It can be coupled with convolutional layers to extend the pixel neighborhood	Vanishing gradient Difficult to train RNN Slow computation time

**Table 3:** A summary of different machine learning evaluation metrics for regression, classification, and image analysis tasks.

Summary of Different Machine Learning Evaluation Metrics		
Regression Metrics	Classification Metrics	Image Analysis
Coefficient of determination (R^2^) Mean squared error (MSE) Root mean squared error (RMSE) Mean absolute error (MAE)	Accuracy Precision and recall F1-score Sensitivity and specificity Receiving Operating Characteristic (ROC) Cohen’s Kappa	Average Precision Mean Average Precision Pixel Accuracy Dice Coefficient Intersection-Over-Union

**Table 4:** Summary of different applications of AI in solid dosage forms.

Applications of AI in Solid Dosages Forms (Since 2015)			
Dosage Forms	Applications	Algorithms	Reference
Tablet	Predicting drug release	ANN, SVM, Ensemble of Regression Trees, and decision tree	[80,81]
	Developing 3D-printed tablets	ANN, self-organizing maps, RF, SVM, and CNN	[65,71,82]
	Detecting tablet defects	CNN, You Only Look Once v5 (YOLOv5)	[83,64,65]
	Estimation of disintegration rate	RF, XGBoost, ANN, and CNN	[79,84]
	Drug particle size inspection	Pattern recognition neural network	[85]
Powders	Process control of powder engineering	ANN	[86]
	Designing dry powder for inhalation	RF, XGBoost, LightGBM, SVM, KNN, ANN, and CNN	[87,88]
	Predicting particle size distribution of spray-dried powder	Unspecified	[89]
	Improving spray-dried powder compatibility	SVM and ANN	[90]
	Predicting the extent of agglomeration	SVM, RF, and partial least squares regression	[91]
Capsules	Identifying capsule defects	KNN, SVM, and CNN	[92]
	Detecting the defects of the pellets within the capsules	SVM	[93]
Granules	Granulation process control	Neuro-fuzzy logic and genetic programming	[94]
	Predicting particle size distribution	ANN, multiple linear regression, and genetic programming	[60]
Solid dispersions	Predicting physical or chemical stabilities	ANN, SVM, RF, LightGBM, KNN, and naïve Bayes	[52,59]
	Predicting dissolution rates and profiles	RF, SVM, LightGBM, and XGBoost	[56,95]

## Data Availability

Not applicable.

## Supplementary Materials

The following supporting information can be downloaded at: https://www.mdpi.com/article/10.3390/pharmaceutics14112257/s1, File S1: Key partnerships between AI and pharmaceutical companies.
